# Further electrode evaluation of the Nova 2 ionised calcium instrument

**DOI:** 10.1155/S1463924681000278

**Published:** 1981

**Authors:** J. A. Fyffe, A. S. Jenkins, H. N. Cohen, Frances J. Dryburgh, Mary D. Gardner

**Affiliations:** 1 University Department of Medicine and Department of Biochemistry Royal Infirmary Castle Street Glasgow G4 0SF UK; 2 Dept. of Biochemistry Yorkhill Hospital Yorkhill Glasgow Scotland


### Further electrode evaluation of the Nova 2 ionised calcium instrument

**J. A. Fyffe** †**, A. S. Jenkins, H. N. Cohen, Frances J. Dryburgh and Mary D. Gardner.**
*University Department of Medicine and Department of Biochemistry, Royal Infirmary, Castle Street, Glasgow, G4 0SF, UK.*

### Introduction

In the original evaluation of the Nova 2 analyser [1] inter-electrode differences were shown which would have necessitated the re-establishment of the ionised calcium reference range each time that the calcium ion-selective electrode was changed.

The manufacturers (Nova Biomedical, Newtown, Mass, USA) have now replaced the electrodes which showed slow and variable response times to serum, and it has been possible to compare these two replacement electrodes with the original electrode (still in service after 14 months). Another electrode, replaced under warranty, and two supplied with a second instrument on loan from the British Agents (American Hospital Supplies, Didcot, Oxon, UK) were also compared with the original electrode. In all a total of six electrodes were evaluated.

### Methods

The methods used were those previously described [1] and followed the IUPAC recommendations [2] as far as possible.

Comparisons were made using two instruments, one of which was always fitted with the original ion selective electrode and the other instrument with each of the other five electrodes in turn. Before analysis of specimens, the electrodes were allowed to come to temperature for a minimum of 15 minutes and the instrument purged. Both instruments then performed the same task at the same time.

Interchange of reference electrodes between instruments did not affect the ion selective electrode response.

Samples were assayed simultaneously on both instruments in triplicate and calculations of the difference of results were performed on the means of these determinations using a pair difference t test. This avoided problems due to storage and/or handling of the specimens [3].

†*Present address: Dept. of Biochemistry, Yorkhill Hospital, Yorkhill, Glasgow.*

### Results

Figure 1 shows the calibration curves from the six electrodes examined. The lines are almost parallel. All electrodes obey the Nernst equation giving a change of approximately 58 millivolts with a tenfold change in calcium concentration. Figure 2 shows the calibration curves from the same electrode taken 9 months apart, these remain parallel and only the position of the line has changed. Table 1 shows the limits of detection for each of the six electrodes. There was no significant difference between electrodes and all limits are well below the expected clinical range. The potentiometric selectivity coefficients for elements known to affect the electrodes are also shown in Table 1. These show no significant difference between electrodes. The results for the electrode which has been used for 14 months have not altered significantly (Table 1). Response curves in aqueous solution are shown in Figure 3 and these have been superimposed upon one another to show that the curve shapes are almost identical. Figure 4 shows response times to serum of the six electrodes also superimposed upon one another. Four of the electrodes show similar results but two show slow response times. One is the original which showed a slow response in the previous study. The other was supplied with the instrument on loan from American Hospital Supplies.

The results of duplicate estimations on serum were examined and the results are shown in Table 2. These show that differences in the results from each of the electrodes examined were significant for two electrodes.

During the period of this study one significant problem occurred, aqueous standards gave the expected results although quality control serum did not. Cleaning and replacing the tubing of the instrument did not rectify the problem. It was corrected by fitting a new reference electrode. The fault was not detected by the instrument's computer system and would have resulted in low (by approximately 0.1 mM) results being reported. It was, however detected by the authors' quality control serum.

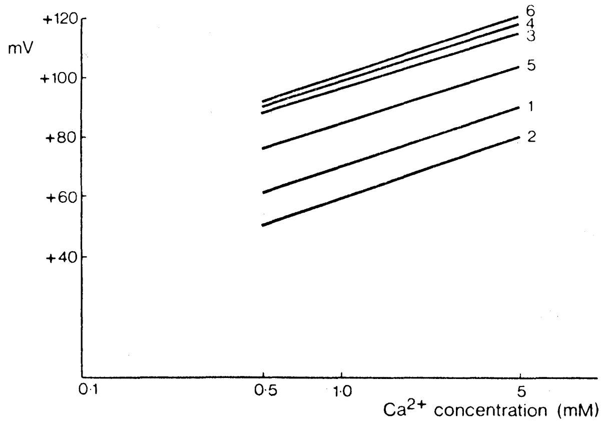

*Figure 1. Calibration curves for the six electrodes examined.*

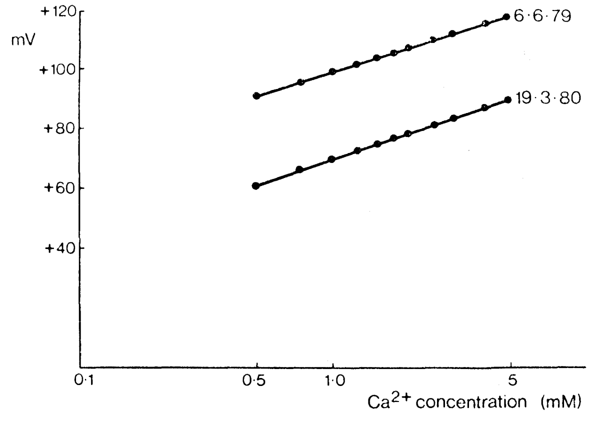

*Figure 2. Calibration curves for the same original electrode nine months apart.*

**Table 1. The potentiometric selectivity coefficients and detection limits for the six electrodes. The results for the original electrode are also shown from 9 months previous.**

| Electrode | $K_{A,B}^{Pot}$ | | | Limit of detection (nM) |
|---|---|---|---|---|
| | Zn (1.5µM) | Mg (0.6mM) | Mg (1.2mM) | |
| 1. (6.6.79) | 42 | 0.16 | 0.06 | 0.020 |
| 1. (19.3.80) | 35 | 0.12 | 0.06 | 0.033 |
| 2. " | 35 | 0.14 | 0.07 | 0.033 |
| 3. " | 52 | 0.13 | 0.10 | 0.037 |
| 4. " | 51 | 0.15 | 0.09 | 0.030 |
| 5. " | 29 | 0.09 | 0.05 | 0.031 |
| 6. " | 37 | 0.12 | 0.08 | 0.026 |

### Discussion

The Nova 2 continues to offer a robust and convenient means of estimating ionised calcium and no significant differences were noted between the six electrodes in the calibration curves, selectivity coefficients and aqueous reponse times. However, response times to serum did show some differences. Four electrodes showed a fast response with a plateau obtained earlier than with the original electrode [1]. There were no significant differences between the four although the response slopes never reached a true plateau as seen with aqueous solutions. One electrode showed a slow response, similar (but not identical) to the original, which the manufacturers described as not meeting their quality control specifications. This electrode was supplied with the instrument on loan from American Hospital Supplies. The inter-electrode comparisons between replicate samples show no significant difference between four of the electrodes including the original. A significant difference for the other two electrodes from the four previously mentioned was noted. Both these were supplied with the instrument on loan from American Hospital Supplies. One also had a slow response time. These results show that a slow response time is not the only cause of differing results with serum.

It is disturbing that the manufacturers continue to issue electrodes with new instruments which have a poor response time when used with serum and different batches can give significantly different results. This necessitates the establishment of a reference interval for use with any one electrode.

The authors recommend that purchasers of the Nova 2 instrument examine the response time of the electrodes supplied before final acceptance and use, or require the manufacturers to produce evidence of satisfactory response for the particular electrode. The authors also recommend that careful checks are made of serum quality control data when electrodes are changed.

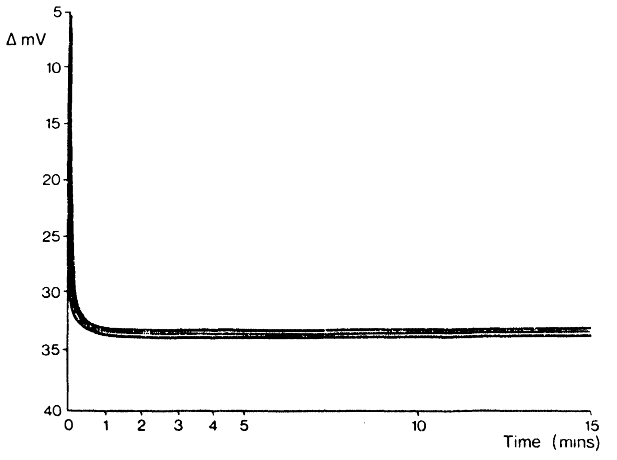

*Figure 3. Response time curves for the six electrodes examined to aqueous solutions.*

**Table 2. The number, mean and standard deviation for comparison estimations on sera by two electrodes. The significance of paired difference t tests are shown alongside each pair of comparisons.**

| Serum Group | Electrode number | n | Mean and SD | p |
|---|---|---|---|---|
| A | 1 | 19 | 1.068<br>0.063 | NS |
| | 2 | | 1.061<br>0.054 | |
| B | 1 | 23 | 1.107<br>0.060 | NS |
| | 3 | | 1.115<br>0.053 | |
| C | 1 | 23 | 1.133<br>0.050 | NS |
| | 4 | | 1.138<br>0.056 | |
| D | 1 | 21 | 1.105<br>0.059 | <0.005 |
| | 5 | | 1.067<br>0.050 | |
| E | 1 | 21 | 1.152<br>0.061 | <0.001 |
| | 6 | | 1.122<br>0.057 | |

It is also recommended that stringent quality control procedures be adopted to avoid problems such as were encountered with the reference electrode where the instrument's "trouble shooting" computer program failed to detect the fault. A spare reference electrode is not supplied with the instrument.

ACKNOWLEDGEMENT

The authors wish to acknowledge the financial support of the Kidney Unit Fund, Glasgow Royal Infirmary, towards the cost of the instrument. The authors also wish to thank Carol Bolland for her technical assistance.

REFERENCES

[1] Fyffe, J. A., Jenkins, A. S., Cohen, H. N., Dryburgh, F. J. and Gardner M. D. *Journal of Automatic Chemistry* (1980) **2**, 85.

[2] IUPAC Information Bulletin, 1978, No. 1 29th General Assembly Council Meeting, Warsaw.

[3] Robertson, W. G. *Annals of Clinical Biochemistry* (1976) **13**, 540.

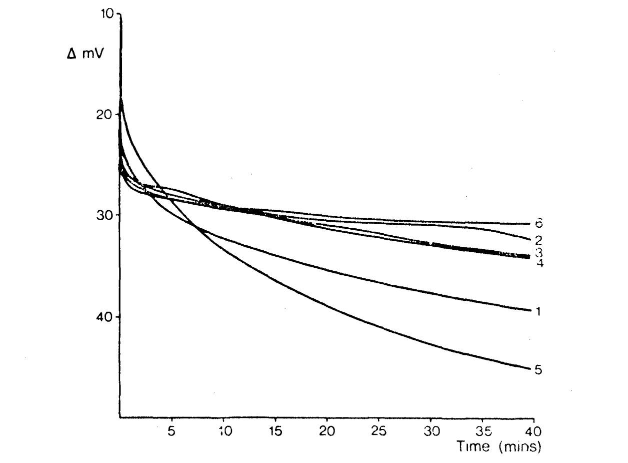

*Figure 4. Response time curves for the six electrodes examined to serum.*

